# Integrating automation and LC/MS for drug discovery bioanalysis

**DOI:** 10.1155/S1463924602000019

**Published:** 2002

**Authors:** David T. Rossi

**Affiliations:** Bioanalytical Core Group Department of Pharmacokinetics Dynamics and Metabolism Pfizer Global Research and Development Ann Arbor MI 48105 USA

## Abstract

A novel, integrated approach for automated sample handling in drug discovery bioanalysis is described. The process includes aspects of animal study design, biological sample collection, sample processing and high-throughput APILC/MS operating in under multiple reaction monitoring (MRM). A semi-automated 96-well liquid—liquid extraction technique for biological fluid sample preparation was developed and used in conjunction with the integrated sample-handling approach. One plate of samples could be prepared within 1.5 h compared with 4 h for a manual approach, and the resulting 96-well plate of extracts was directly compatible with the LC/MS. Feasibility studies for the development of the process included sample collection map generation and information management, sample collection formatting, evaluation of alternative dilution schemes for high-concentration samples, choice of biological fluid, and evaluating the capabilities of the two liquid-handling workstations. Numerous comparisons between the new approach and conventional sample-handling approaches gave equivalent drug-quantitation results for several example compounds. This new sampling process has approximately doubled the efficiency (as measured by studies assayed per month) of drug discovery bioanalysis in our laboratory. The approach was also used in conjunction with time-of-flight mass spectrometry instrumentation (LC/TOF/MS) to quantify and characterize the disposition of simultaneously dosed example drug compounds in the rat. Likely strategies for future automated sample preparation workstations are described.


Journal of Automated Methods & Management in Chemistry
Vol. 24, No. 1 (January–February 2002) pp. 1–7

### Integrating automation and LC/MS for drug discovery bioanalysis†

**David T. Rossi**

*Bioanalytical Core Group, Department of Pharmacokinetics, Dynamics and Metabolism, Pfizer Global Research and Development, Ann Arbor, MI 48105, USA*

*A novel, integrated approach for automated sample handling in drug discovery bioanalysis is described. The process includes aspects of animal study design, biological sample collection, sample processing and high-throughput APILC/MS operating in under multiple reaction monitoring (MRM). A semi-automated 96-well liquid–liquid extraction technique for biological fluid sample preparation was developed and used in conjunction with the integrated sample-handling approach. One plate of samples could be prepared within 1.5 h compared with 4 h for a manual approach, and the resulting 96-well plate of extracts was directly compatible with the LC/MS. Feasibility studies for the development of the process included sample collection map generation and information management, sample collection formatting, evaluation of alternative dilution schemes for high-concentration samples, choice of biological fluid, and evaluating the capabilities of the two liquid-handling workstations. Numerous comparisons between the new approach and conventional sample-handling approaches gave equivalent drug-quantitation results for several example compounds. This new sampling process has approximately doubled the efficiency (as measured by studies assayed per month) of drug discovery bioanalysis in our laboratory. The approach was also used in conjunction with time-of-flight mass spectrometry instrumentation (LC/TOF/MS) to quantify and characterize the disposition of simultaneously dosed example drug compounds in the rat. Likely strategies for future automated sample preparation workstations are described.*

### Introduction

Emerging technologies such as combinatorial chemistry [1, 2] and cassette dosing [3, 4] have accelerated the drug-discovery process. In response, the demand for bioanalytical practice has resulted in faster analytical techniques and higher preparation capacity. At present, one of the most widely used techniques in bioanalytical laboratories supporting pharmacokinetics, drug transport and metabolism is liquid chromatography-tandem mass spectrometry (LC/MS/MS) with mutiple-channel-monitoring (MRM) [5, 6]. The technique has greatly facilitated bioanalytical work, so that the most time-consuming and labour-intensive steps are still associated with sample handling. Recent studies have reported significant improvements in the sample-preparation process. One area showing much progress is the automation of sample preparation by using various liquid-handling workstations. These workstations replaced manual liquid transfers and use parallel sample preparation (e.g. 96-well plate) for higher sample throughput [7–9]. Workstations have proven useful by improving the sample preparation process through more effective extractions [10], better automation of extractions [11], reduction in scale [12] and use of automated method development [13]. These advances are reducing method development time and, in some aspects, increasing assay quality. These efforts, however, only focus on the sample preparation step of the bioanalytical process. Other than those sampling approaches utilized for on-line, *in vivo* microdialysis sampling [14], there appears to be no recent papers describing a comprehensive sample handling for the drug-discovery process.

In drug discovery, processes closely associated with bioanalysis (BA) are study design and animal study conduct/sample collection. These three processes are often driven by groups operating sequentially but independently, or by equivalent, separate groups operating in parallel. Lack of integration or standardization in sample collection and information delivery present in these processes can result in inefficiency for sample collection/processing and break-down in information between the three groups. For example, an analytical chemist could spend considerable time transferring samples to a 96-well format before automated processing on a liquid-handling workstation. At this point, it seems reasonable that greater process efficiency could be attained if a closer relationship between the separate processes of study design, sample generation and bioanalytical sample preparation were developed.

This paper reports on an integrated sample-handling process for bioanalytical discovery, based on biological sample collection directly in a 96-well format and taking into account the front-end preclinical protocol design and sample-processing requirements. The purpose was to demonstrate standardized and efficient sample-handling procedures for drug discovery. A comparison of different sample collection formats and sample-processing options is also reported. Characteristic pharmacokinetic data obtained from the different approaches are also discussed and compared for four example compounds.

### Experimental

### *Analyte test system, reagents and LC/MS experimental conditions*

Analytes and reagents, such as drugs, organic solvents, blank rat plasma and HPLC mobile phase, were purchased or prepared from the same sources as those in Zhang *et al.* [15]. Briefly, diphenhydramine, desipramine, chlorpheniramine and trimipramine were the four compounds tested here. Lidocain was added as an internal standard. The same LC/MS apparatus and sample

† This paper was initially presented at the ISLAR 2000 Conference and is reproduced here by kind permission of Zymark Corporation.

preparation conditions as those in [15] were also used here.

*Apparatus*

A MultiProbe II (Packard Instruments, Meriden, CT, USA) was used to transfer plasma and serum. It was equipped with an $x, y, z$-coordinate robotic arm with four sampling tips. It was optimized for aspiration and dispensation of different liquids with varying viscosities. Small conductive disposable tips were used together with the liquid-sensing function when aspirating and dispensing (3 mm below the liquid surface). A Tomtec Quadra 96 model 320 (Hamden, CT, USA) was used to handle all 96-well parallel-liquid transfers such as internal standard addition, organic solvent addition, organic layer transfers and reconstitution after nitrogen dry down. This semi-automated 96-well liquid-extraction approach using a Tomtec workstation was introduced in [15]. An Eppendorf centrifuge (model 5810R, Hamburg, Germany) operating at 3000 rpms was a refrigerated bench-top centrifuge that could accommodate 96-well plates.

*Drug administration and sample collection*

Two studies were conducted to evaluate potential strategies for integration of the sample collection step with sample processing. In each experiment, six male Wistar rats (three for oral, three for intravenous) were dosed. The animals were fasted for 12 h before drug administration. Drugs I–IV were dissolved in a 10% ethanol aqueous solution (4 mg ml$^{-1}$) for oral gavage. Intravenous infusion solutions contained 4 mg ml$^{-1}$ of each drug dissolved in a mixture of ethanol:dextrose (5%) aqueous solution (10:90 v/v). The total dose was 10 mg kg$^{-1}$ for both oral and intravenous treatments. The time points for collection in each study were predose, 30 min, 1 h, 2, 4 and 6 h. Whole-blood samples were collected in either serum or plasma (with sodium heparin) separator tubes. Samples were placed directly into individual 1.1-ml polypropylene tubes in a 96-well tube-rack format (Costar, Cambridge, MA, USA). The arrangement of the sample tubes in the rack will be discussed below.

For the first study, two plates of plasma samples were collected with ~ 400 µl blood at each time point. In the first plate, exact plasma volumes of 25 µl (30 min and 1 h samples) or 100 µl (predose, 2 h, 4 and 6 h) were transferred to tubes in the 96-well plate rack. To the second plate, the remaining volumes of ~ 120–220 µl were transferred and frozen (−20°C).

For the second study, three rats were dosed intravenously with the same four drugs. Both serum and plasma samples were harvested from the same animal, transferred into respective 96-well tube plates and frozen (−20°C) for quantitative comparison at a later time.

Pharmacokinetic parameters were calculated using WinNonlin software (v. 2.1, Pharsight Corporation, Palo Alto, CA, USA).

*Experimental overview*

*Multiprobe precision assessment.* Testing the precision of liquid transfer by the MultiProbe II was performed for plasma and serum. Assessment involved gravimetric determinations of transfer volumes.

*Parallelism assessment.* To evalute the effect of different dilution approaches on quantitation, blank rat plasma was spiked with the four drugs at 1000 ng ml$^{-1}$. Several dilution approaches were evaluated, including direct assay of 100 µl plasma, direct assay of 25 µl plasma (dilution factor of 4) and 25 µl spiked plasma plus 75 µl blank plasma (dilution factor of 4). These synthetic samples were transferred into the 1.1-ml polypropylene tubes in 96-rack format by manual transfer and assayed ($n = 3$) for a comparative assessment of parallelism.

### Results and discussion

*Description of integrated sample handling process*

A pharmacokinetic cassette study typically consists of a dosing solution of three to six compounds that are dosed orally in three animals and intravenously in three other animals. The total number of samples generated is 30–60 depending on the number of time points desired. The time spent in labelling, decapping and transferring samples to alternate formats is often significant. Use of the 96-well format can drastically decrease the need for these steps, so it seems likely that integration of study design, sample collection and bioanalytical sample preparation would improve the efficiency of the preclinical drug discovery process.

Although automatic liquid-handling workstations now replace manual transfer [7–9], they required a standard sample format such as 96- or 384 wells for maximum efficiency [13]. Traditionally, in drug disposition studies, biological fluids (plasma, serum, urine, etc.) are collected in individual, capped tubes or bottles. Each tube is labelled individually with the collection time and animal number before dosing. The volumes of samples are varied. To work around the transfer of biological fluid samples from individual tubes to 96-well plates, it is possible either to: (1) perform manual transferal of samples, (2) use a liquid-handling workstation to transfer samples or (3) generate initially and deliver samples directly to a 96-well format. In our laboratories, each approach has been used to some extent, with manual transferal being the least desirable. The use of a liquid-handling workstation such as the Packard Multiprobe has shown some utility in transferring samples from individual vials to a 96-well plate. Most recently, however, we have adopted the latter approach: the initial generation and delivery of samples directly in a 96-well plate. Although not applicable to all types of sample matrices, most notably tissue samples, this approach can streamline the sample-preparation approach by eliminating one or more sample transfer steps and by allowing 96-well sample preparation to proceed more efficiently.

Because 96 wells is a universal format that fits various automatic workstations, it can be built into those steps preceding bioanalysis, including study design and sample collection. Figure 1 depicts an integrated sample/information flow for a typical discovery phase *ex vivo* experiment such as a cassette-dosing experiment. In this scheme, a discovery scientist designs a protocol and builds a sample list that is sent electronically to an animal models group. The animal models group executes the dosing protocol, collects and delivers samples to a 96-well plate along a predefined plate map (figure 2). At this point, some of the sample locations in the 96-well plate remain vacant to accommodate standards and controls which will be added later. From this point, the samples remain in the 96-well format, although they may be frozen, thawed, centrifuged, automatically transferred or otherwise processed in parallel by the bioanalytical chemist. After sample processing, a 96-well plate is delivered to an autosampler and injected into the LC/MS/MS system for separation, detection and quantitation. Quantitation results are reported to a pharmacokineticist in a format that has been previously defined by the sample list and study protocol. This approach offers a cogent, streamlined approach to sample collection and data handling for most discovery-phase experiments. Similar processes have been created for the collection of CACO-2 and other *in vitro* experiments.

The 96-well map (figure 2) is recommended here for any study where samples are to be harvested. If designed correctly, this map can provide a blueprint or plan for study design, sample collection, sample assay, data reporting and data-processing structures. The standard 96-well plate has eight rows, each containing 12 wells in each row. In our model, samples from orally dosed rats [1–3] were arranged in rows A–C. Samples from the three intravenously dosed rats [4–6] were arranged in rows D–F. In each row, time points were assigned (e.g. first well for predose, second well for 15-min collection, third well for 30-min collection, etc.). The collection volume may also be labelled in the map or in an accompanying spreadsheet. The 12 wells in rows G and H are reserved for standards, blanks or quality controls as needed.

Although the 96-well or similar format is essential to the plan, 1.1-ml tubes were selected for containment of individual samples. The first consideration behind this decision was sample stability: samples can be capped and placed into a freezer in the 96-well format immediately after each time-point is collected. Without individual tubes, an entire plate would need to be put in and pulled out of the freezer repeatedly. Any associated freezing or thawing could cause analyte degradation and clot formation in plasma.

A second consideration, as detailed in [15], is the ability to do automated or semi-automated extraction directly within the 96-well format. Liquid extraction is especially facilitated by individual tubes containing samples. A 96-well plate containing such tubes is placed in one stage of the Tomtec and a semi-automated liquid–liquid extraction is performed. In this strategy, no sample format conversion, labelling or randomization is necessary so that the time required for bioanalysis is greatly improved. In this way, a higher degree of integration and streamlining of the *in vivo* portion of the drug-discovery process is achieved.

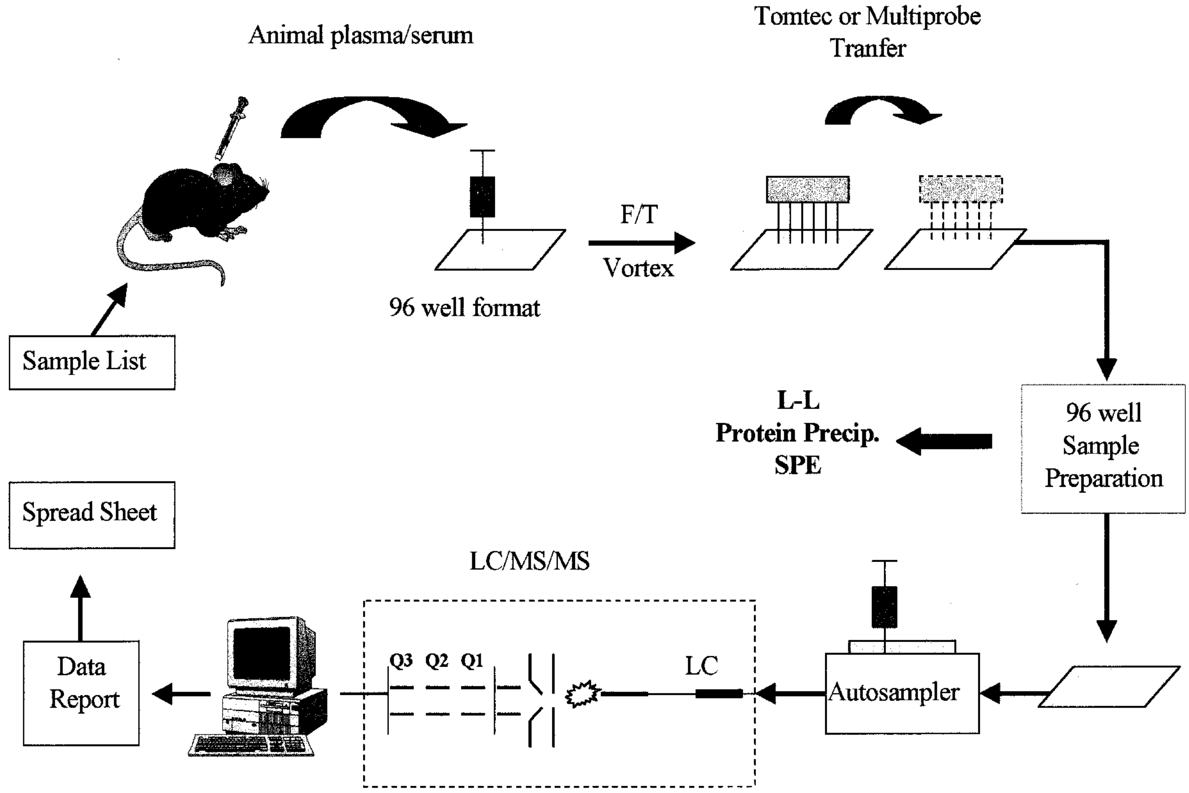

*Figure 1. Conceptual diagram of the integrated sample-handling process for discovery bioanalysis.*

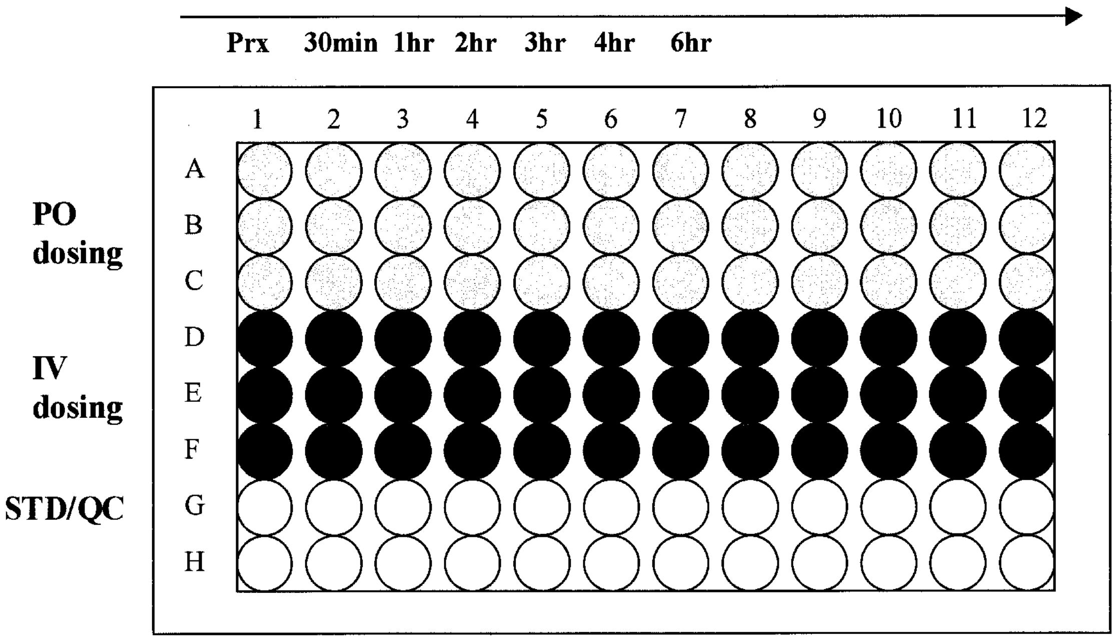

*Figure 2. Predefined 96-well plate map, which can give details of dosing protocol and sample collection.*

*Parallelism studies*

High level samples (15, 30 min and 1 h) need to be diluted before assay. The precision and accuracy of several dilution methods were compared. The standard curve was constructed from 1 to 2000 nm $mg^{-1}$ and a relatively high concentration (1000 ng $ml^{-1}$ of drugs I–IV) was chosen for the test. In the first approach, 100 µl plasma was directly assayed without dilution. In the second approach, 25 µl plasma was directly extracted and a dilution factor of 4 was used. In the third approach, 75 µl blank plasma was added to 25 µl of each plasma sample and a dilution factor of 4 was again used. In each case, three replicates were tested. The results (table 1) suggest that the three approaches yielded results that were statistically indistinguishable and gave acceptable precision (< 2% RSD, except drug IV at 100 µl straight) and accuracy (generally < 7.2% relative error, except for drug IV at 25 + 75µl blank). The second approach is commonly used in our laboratory because it offers equal performance with a smaller number of dilution steps. The reliability of this approach appears to be excellent, while volume aliquoting is reduced. This experiment has practical importance in terms of simplifying the analytical procedure and in providing guidance in the exact volume of sample necessary for collection. It seems likely that using a reduced sample volume without diluting plasma will be useful for other compounds in similar matrices.

*Quantitative comparison of exact-volume-delivered samples and volume-transferred samples*

To convert samples to a 96-well format automatically, a MultiProbe II or similar workstation is commonly used because of its flexibility, good precision and accuracy. An evaluation of the performance of transferring biological samples such as plasma and serum was conducted to estimate precision and accuracy. Twelve individual tubes were weighted before and after transfer of 100 µl plasma or serum. The imprecision associated with these transfers was < 0.5% RSD for rat plasma using the multiprobe after brief centrifugation. The imprecision (RSD%) for manual transfers was 2% RSD.

A comparison study between the collection of exact volumes of plasma and the collection of unknown (total) volumes of plasma was performed. For the exact-volume experiment, volumes of plasma were collected (25 µl at 30 min and 1 h, 100 µl at other time-

*Table 1. Quantitative comparison (n= 3) of three dilution approaches for high concentration sample assay.*

| | *100 µl, no blank plasma added* | | | *25 µl, no blank plasma added* | | | *25 µl + 75 µl blank plasma* | | |
|---|---|---|---|---|---|---|---|---|---|
| *Compound** | *Conc.* | *RSD%* | *RE%* | *Conc.* | *RSD%* | *RE%* | *Conc.* | *RSD%* | *RE%* |
| I | 1023 | 0.5 | 2.3 | 928 | 1.1 | −7.2 | 975 | 1.7 | −2.5 |
| II | 1013 | 1.7 | 1.3 | 1020 | 1.6 | 2.0 | 1049 | 1.4 | 4.9 |
| III | 1007 | 0.8 | 0.7 | 892 | 0.6 | −10 | 954 | 1.4 | −4.6 |
| IV | 977 | 5.6 | −2.3 | 1056 | 1.3 | 5.6 | 808 | 1.1 | −19 |

* I, dephenhydramine; II, desipramine; III, chlorpheniramine; IV, trimipramine. Samples contained 1000 ng $ml^{-1}$ of each compound spiked into blank plasma.

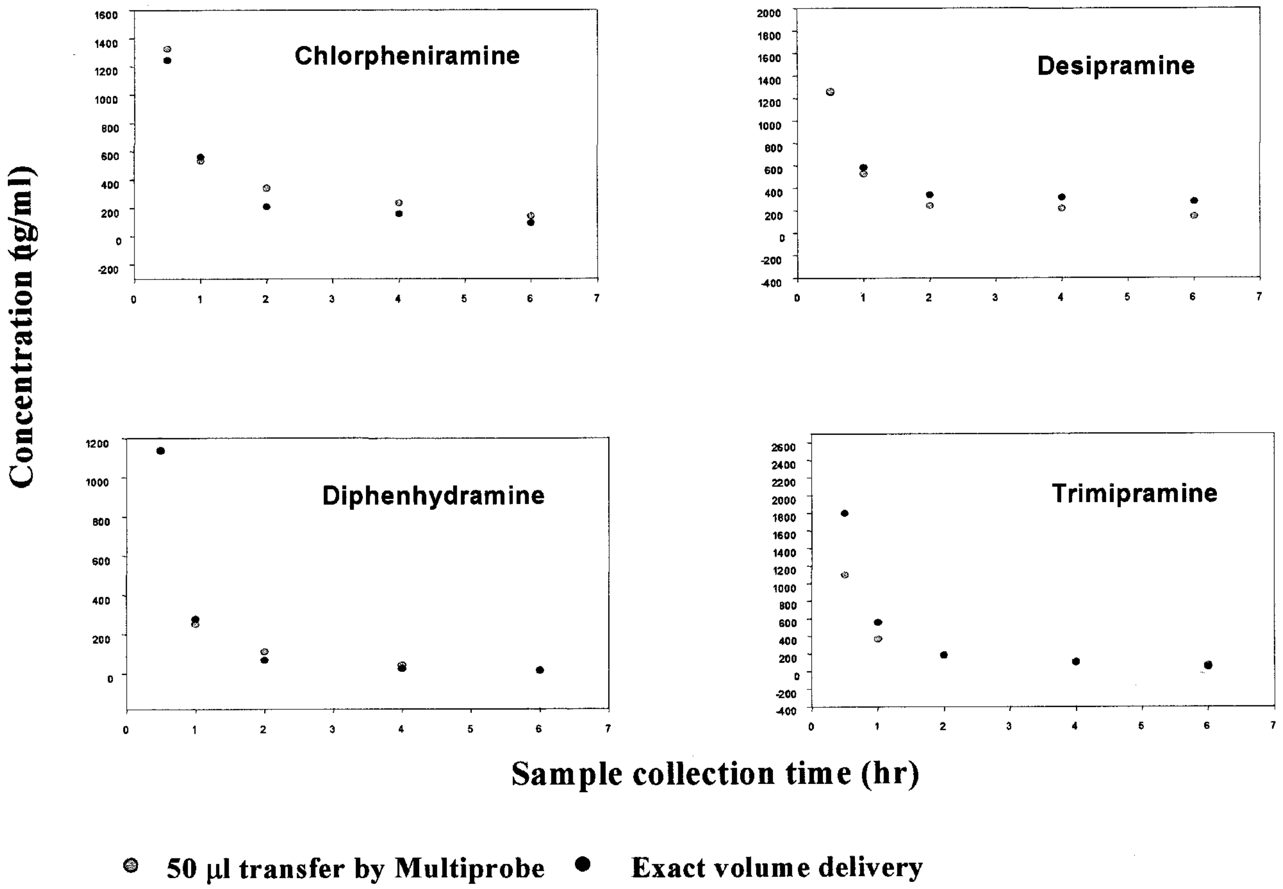

*Figure 3. Comparison of drug concentrations for four example compounds after sample processing by two approaches: 1, exact volumes of plasma delivered to the sample collection tubes in a 96-tube rack; 2, post-collection transfer of plasma to a 96-tube rack by MultiProbe.*

points) into a 96-well tube rack. In the total-volume approach, ~ 200 µl plasma was harvested and stored in the 96-well tube rack format. Subsequently, 50 µl of each sample was transferred by MultiProbe from the original collection vessel to a new 96-well tube rack. To compare the results of these different procedures, the post-dose concentrations of drugs I–IV were determined (figure 3). The same pharmacokinetic profile was obtained for each example compound. Differences in plasma levels between each of the four compounds ranged from 0.5 to 40%, but averaged < 12.5%. The high degree of similarity in concentrations obtained by the two approaches substantiates the reliability and applicability of the integrated sample-handling process.

*Brief comparison of serum and plasma*

A comparison of the sample-handling properties between serum and plasma was made for human and non-human *ex vivo* samples. Results of transfers using fresh serum or heparinized plasma were comparable: no clots were visible and none were detected with the clot-sensing mechanism. All transfers were successful, with variance of < 1% for 100-µl aliquots. Upon one freeze–thaw (FT) cycle, the sera remained clear, whereas all plasma tubes contained some turbidity. Visual inspection showed that many plasma tubes also contained some clotted material. Statistical evaluation of 100-µl transfers of all plasma and sera, both fresh and frozen, produced similar precision ($n = 47$ each, %RSD = 1.64% and 0.92%, respectively). However, an important factor was the failure rate for transfers. All transfers with sera were successful regardless of the fresh or frozen history of the samples. Transfers of frozen plasma samples from five of six patients contained clots that were detected by the clot sensor of the Multiprobe II. In some cases, the clotted samples were transferred successfully after several attempts. However, even when transfers of clotted samples were successful, precision sometimes was adversely affected. One of the most important findings of these studies was that when sample volume was limited, the presence of clots had the potential for a significant effect on precision and accuracy of automated transfers.

In contrast, transfers of all previously frozen plasma samples in which sample volume was not limited were successful when a centrifugation (14 000 rpm) step was performed before the transfer. Precision and success rates using centrifuged plasmas were comparable with those of sera. Centrifugation did not provide additional improvement for the transfer of sera.

To evaluate the drug concentrations in serum and plasma, both matrices were collected at all time-points of the study protocol. The comparison of serum and plasma concentrations at each time point is shown in figure 4. The average differences in the four compounds

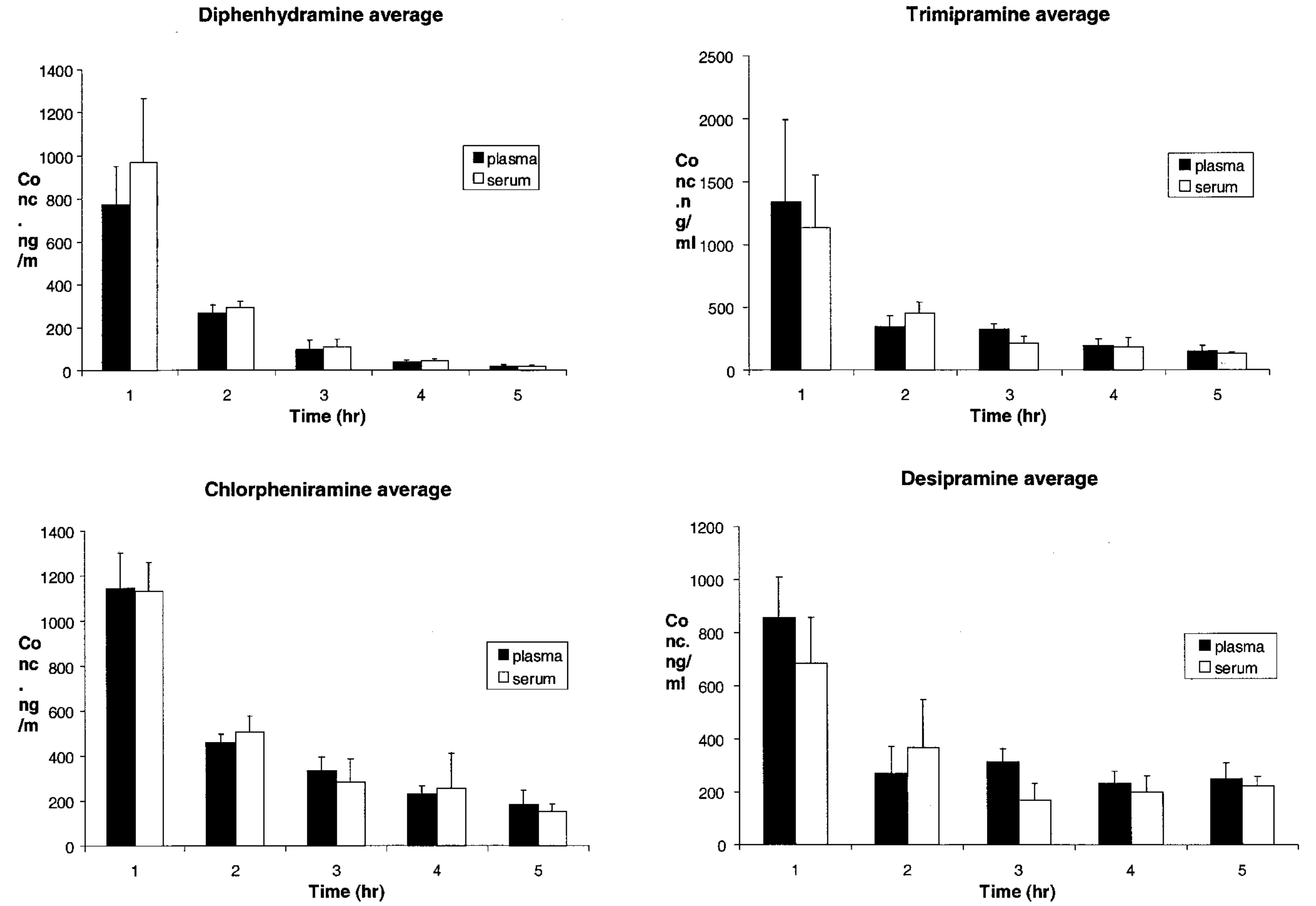

*Figure 4. Comparison of analyte concentrations in plasma and serum samples. Plasma and serum samples were collected from the same animal. The average concentrations of three animals at different time points are shown, where error bars are the SD among the three animals.*

between serum and plasma were: diphenhydramine, 7.2%; desipramine, 22%; chlorpheniramine, 3.3%; and trimipramine, 14%. The results indicate that concentrations from serum and plasma were equivalent to within the limits of experimental variability ($n = 3$). Concentration-related pharmacokinetic parameters obtained from serum and plasma (table 2) agreed well. Because of the limited time-points, other pharmacokinetic parameters such as $t_{1/2}$ were not compared.

In addition to the concern about clots in plasma, another issue was raised during the study. Lipid layers were frequently observed as an upper layer on samples, which affected the experimental results. This issue was addressed by using the MultiProbe II to control the transfer tip at the surface of the sample. A liquid sensor first identified contact with the sample surface, then the program controlled the tip descent to an additional specified distance. Plasma samples from some high-fat rabbit plasma samples produced a considerable (25–50% of the volume) layer upon standing for several hours at room temperature. Lipids could be handled by directing the tip through the top layer, but this distance would have to be determined by inspection of the operator as the clot or liquid detection was not effective at differentiating a lipid layer.

*Table 2. Characteristic pharmacokinetics parameters comparison from plasma and serum.*

| | *Bioavailability (%)* | | $C_{max}$ *(ng ml$^{-1}$)* | |
|---|---|---|---|---|
| *Test compound* | *Plasma* | *Serum* | *Plasma* | *Serum* |
| Chlorpheniramine (I) | $31 \pm 4.1$ | $31 \pm 4.7$ | $233 \pm 12$ | $260 \pm 42.7$ |
| Desipramine (II) | $26 \pm 10.4$ | $26 \pm 5.4$ | $184 \pm 54$ | $210 \pm 40$ |
| Diphenhydramine (III) | $1.6 \pm 0.66$ | $1.7 \pm 0.4$ | $16 \pm 2.4$ | $16 \pm 4.3$ |
| Trimipramine (IV) | $3.9 \pm 2.1$ | $3.7 \pm 0.3$ | $27 \pm 12.9$ | $29 \pm 8.6$ |

Data are the average of three rats ± SD of three rats. Data for plasma and serum were obtained from different doses.

Although the issue about the lipid layer in centrifuged plasma can be addressed, a caveat appears to be the need to identify the minimum distance for additional tip movement. This is a particular issue in cases where sample volume is limited because a maximum distance cannot be used by default. If the tip descends too far into the sample after sensing the liquid level, it could disturb a centrifuged clot, thus causing an error.

### References

1. Obrecht, D. and Villalqordo, J. M., 1998, *Solid-Supported Combinatorial and Parallel Synthesis of Small-Molecular-Weight Compound Library* (New York: Pergamon).
2. Terrett, N. K., 1998, *Combinatorial Chemistry* (Oxford: Oxford University Press).
3. Beaudry, F., Le-Blanc, J. C., Coutu, M. and Brown, N. K., 1998, *Rapid Communicatiions in Mass Spectrometry*, **12**, 1216.
4. Shaffer, J. E., Adkison, K. K., Halm, K., Hedeen, K. and Berman, J., 1999, *Journal of Pharmaceutical Sciences*, **88**, 313.
5. Olah, T. V., McLoughlin, D. A. and Gilbert, J. D., 1997, *Rapid Communications in Spectrometry*, **11**, 17.
6. Berman, J., Halm, K., Adkison, K. and Shaffer, J., 1997, *Medicinal Chemistry*, **40**, 827.
7. Parker, T. D., Surendran, N., Stewart, B. H. and Rossi, D. T., 1998, *Journal of Pharmaceutical and Biomedical Analysis*, **17**, 851.
8. Huang, H., Kagel, J. R. and Rossi, D. T., 1999, *Journal of Pharmaceutical and Biomedical Analysis*, **19**, 613.
9. Simpson, H., Berthemy, A., Burhman. D., Burton, R., Newton, J., Kealy, M., Wells, D. and Wu, D., 1998, *Rapid Communications in Spectrometry*, 75.
10. Bentley, M. C., Abrar, M., Kelk, M., Cook, J. and Phillips, K., 1999, *Journal of Chromatography B Biomedical Science and Applications*, **723**, 185.
11. Diamond, F. X., Vickery, W. E. and de-Kanel, J, 1996, *Journal of Analytical Toxicology*, **20**, 587.
12. Henion, J., Brewer, E. and Rule, G. S., 1999, *Today's Chemistry at Work*, February, 36.
13. Rossi, D. T., 1999, *Trends and Applications in Bioanalysis, LG-GC*, **17**, s4.
14. Elmquist, W. F. and Sawchuk, R. J., 1997, *Pharmaceutical Research*, **14**, 267.
15. Zhang, N., Hoffman, K. L., Li, W. and Rossi, D. T., 2000, *Journal of Pharmacology and Biomedical Analysis* (in press).

